# Receptors of intermediates of carbohydrate metabolism, GPR91 and GPR99, mediate axon growth

**DOI:** 10.1371/journal.pbio.2003619

**Published:** 2018-05-17

**Authors:** Hosni Cherif, François Duhamel, Bruno Cécyre, Alex Bouchard, Ariane Quintal, Sylvain Chemtob, Jean-François Bouchard

**Affiliations:** 1 School of Optometry, Université de Montréal, Montreal, Quebec, Canada; 2 Department of Pediatrics, Research Center-CHU Sainte-Justine, Montreal, Quebec, Canada; 3 Department of Pharmacology, Université de Montréal, Montreal, Quebec, Canada; University of Cambridge, United Kingdom of Great Britain and Northern Ireland

## Abstract

During the development of the visual system, high levels of energy are expended propelling axons from the retina to the brain. However, the role of intermediates of carbohydrate metabolism in the development of the visual system has been overlooked. Here, we report that the carbohydrate metabolites succinate and α-ketoglutarate (α-KG) and their respective receptor—GPR91 and GPR99—are involved in modulating retinal ganglion cell (RGC) projections toward the thalamus during visual system development. Using ex vivo and in vivo approaches, combined with pharmacological and genetic analyses, we revealed that GPR91 and GPR99 are expressed on axons of developing RGCs and have complementary roles during RGC axon growth in an extracellular signal–regulated kinases 1 and 2 (ERK_1/2_)-dependent manner. However, they have no effects on axon guidance. These findings suggest an important role for these receptors during the establishment of the visual system and provide a foundational link between carbohydrate metabolism and axon growth.

## Author summary

Development of the visual system requires high levels of energy and tight regulation of multiple factors integrated by axon projections during navigation to their appropriate targets. While intermediates of carbohydrate metabolism have key roles in many biological processes, much less is known about their effects on receptors in the developing nervous system. We hypothesized that activation of two G-protein-coupled receptors (GPCRs) by metabolic intermediates could promote growth during retinal ganglion cell (RGC) axon extension and guidance from the retina to the brain. We first demonstrated that receptors for two intermediates of carbohydrate metabolism—succinate and α-ketoglutarate (α-KG)—are expressed on developing RGCs and their projections. We revealed that these receptors have a complementary role in regulating axon growth in an extracellular signal–regulated kinases 1 and 2 (ERK_1/2_)-dependent manner, although with no effect on axon guidance. The absence of either receptor caused a strong decline in axonal projections from the retina to the thalamus, while the combined absence of both receptors had an additive effect. Taken together, our findings indicate, for the first time, an important role for intermediates of carbohydrate metabolism and their receptors in stimulating axon growth during the establishment of the visual system and suggest a wider involvement in the nervous system development.

## Introduction

GPR91 and GPR99 are G-protein-coupled receptors (GPCRs) activated by Krebs cycle intermediates, part of the larger class of carbohydrate metabolites—an observation that renewed interest in a biochemical pathway discovered decades ago [1,2]. GPR91, through its activation by succinate outside the tricarboxylic acid (TCA) cycle, has a wide range of functions in diverse diseases, such as hypertension and diabetes. Its study allowed greater understanding of the molecular links between the TCA cycle and metabolic diseases [2,3].

The development of the visual system requires high levels of energy to propel mitochondrial-enriched axons properly through the nervous system, as retinal ganglion cells (RGCs) are essential for transmitting information from the retina to the brain. The growth and survival of neurons depend on mitochondria as they perform aerobic ATP synthesis and play a significant role in apoptotic and necrotic cell death [4]. Thus, failures of mitochondrial function appear to be involved in degenerative diseases of the nervous system [5]. One of the most mitochondria-enriched regions of the axon is the active growth cone (GC) at the tip of the axon [6]. The GC contains multiple receptors that interact with guidance molecules, allowing the front end of a developing axon to navigate through the complex landscape of the early nervous system toward its appropriate targets [7]. However, the role of intermediates from carbohydrate metabolism during the development of the visual system has not been well characterized.

In the past decade, increasing evidence has highlighted GPCRs as mediators of both repulsive and attractive axon guidance, as their ligands may serve as guidance cues for axon pathfinding; however, GPCRs involved in axon growth still remain to be found [8–11]. In a groundbreaking study in 2004, GPR91 (succinate receptor 1 [Sucnr1]) and GPR99 (2-oxoglutarate receptor 1 [Oxgr1]) were both identified as receptors of the Krebs cycle intermediates succinate and α-ketoglutarate (α-KG), respectively [2]. GPR91 and the closely related GPR99 are expressed in multiple tissues, such as the kidney [2,12] and cardiac muscle [13–15]. Previous reports have shown that succinate and GPR91 regulate normal retinal vascularization, proliferative ischemic retinopathy [16], and cortical revascularization post-ischemia [17]. Moreover, through the activation of GPR91, succinate has been shown to have an effect on motility, migration, and growth, as it directly promotes chemotaxis and potentiates activation initiated by Toll-like receptor agonists in dendritic cells [18,19]. However, to date, scarce literature exists on GPR99 functions.

Human neuronal mapping and vascular innervation are closely related, as similar molecules and signaling mechanisms are shared between axon guidance, neuronal migration, and blood vessel guidance and growth. For example, the Slit/Robo pathway plays a critical role in both angiogenesis and the guidance of neuronal migration of the olfactory system [20,21]. Moreover, semaphorins and their receptors play a pivotal role as axon guidance cues [22,23] while also acting as a vasorepulsive force that misdirects new retinal vessels toward the vitreous in a murine model of oxygen-induced retinopathy [24].

Therefore, we investigated the growth-promoting actions and guidance effects of the carbohydrate metabolites succinate and α-KG, through their respective receptor GPR91 and GPR99, during the establishment of the retino-thalamic pathway in an embryonic mouse model. Elucidating carbohydrate metabolite functions during visual development may provide crucial insights regarding their potential roles in the plasticity and regeneration of the nervous system and allow the development of further pharmacological tools, expanding and improving central and peripheral nervous system repair strategies.

## Results

### GPR91 and GPR99 are expressed in RGCs in the developing retina

**Fig 1:**
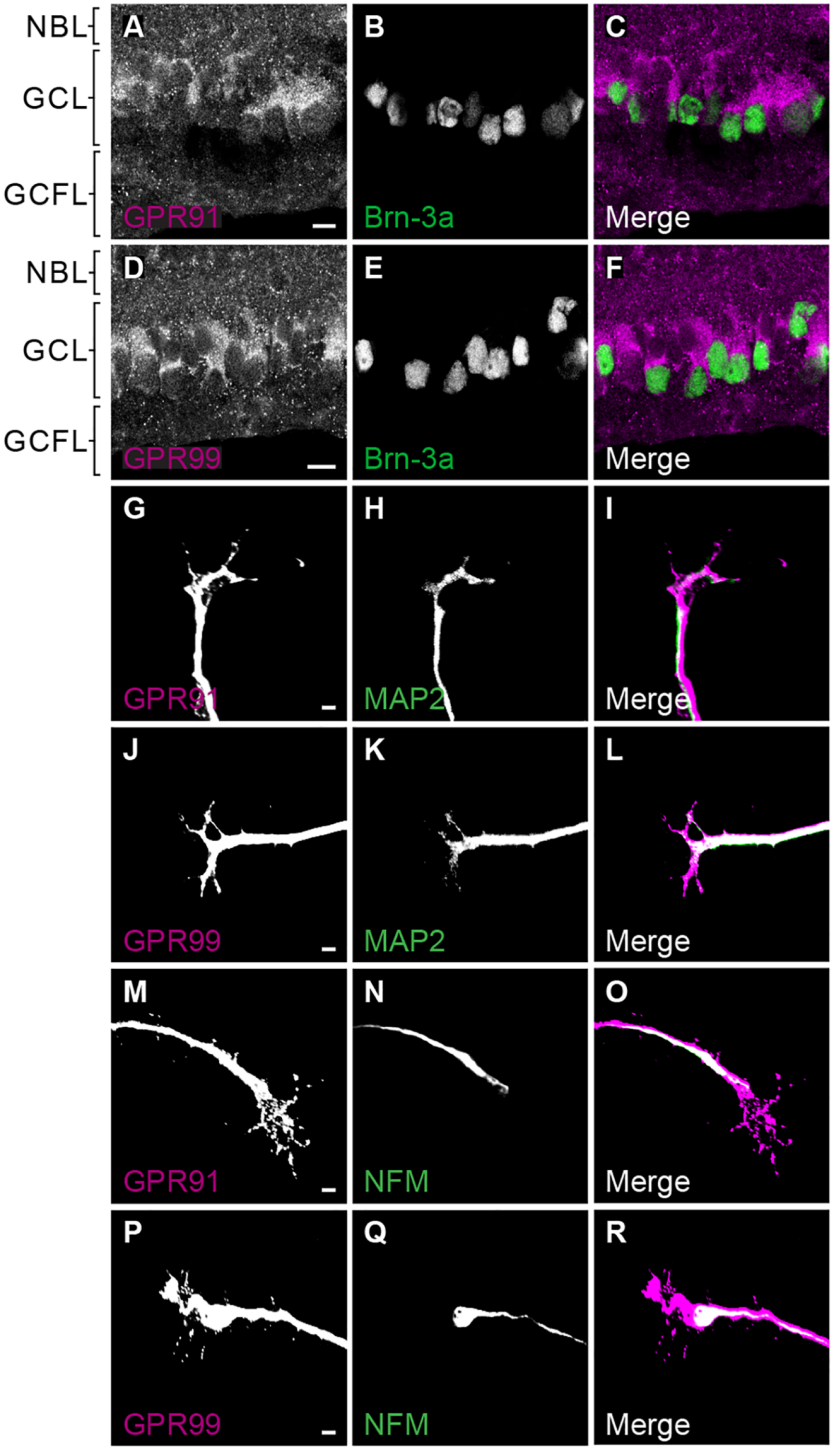
GPR91 and GPR99 are expressed in RCGs and in axons and dendrites of retinal explants. **(A-F)** Expression of GPR91 and GPR99 proteins in retinal sections of E14/15 WT mouse embryos. Scale bars: 10 μm. **(G-R)**: Expression of GPR91 and GPR99 in retinal explants, GCs, and neurites of E14/15 WT mouse embryos. Double-labeling immunofluorescence illustrates the colocalization of GPR91 and GPR99 with dendrites or with axons of RGCs. Scale bars: 5 μm. E14/15, embryonic day 14/15; GC, growth cone; GCFL, ganglion cell fiber layer; GCL, ganglion cell layer; MAP2, microtubule-associated protein 2; NBL, neuroblast layer; NFM, neurofilament; RGC, retinal ganglion cell; WT, wild-type.

We utilized murine retinas obtained from embryos (embryonic day 14/15 [E14/15]) to characterize the presence of GPR91 and GPR99 and their possible involvement during retinal projection navigation. At E14/15, GPR91 and GPR99 proteins were mainly present in the ganglion cell layer but were also detected in the ganglion cell fiber and neuroblast layers (Fig 1A–1F). The retinas from adult and E14/15 knockout (KO) mice (*gpr91*KO or *gpr99*KO) showed no expression of GPR91 or GPR99, confirming the antibodies’ specificity (S1A–S1H Fig). In E14/15 wild-type (WT) murine retinal explants, GPR91 and GPR99 were present in neurites, GCs, and filopodia, in dendrites and axons (Fig 1G–1R and S1I–S1L Fig). Retinal explants obtained from *gpr91*KO and *gpr99*KO E14/15 embryos did not express GPR91 or GPR99, respectively (S1M–S1P Fig), which also confirms the specificity of the antibodies used in this study. Moreover, we observed the presence of GPR91 and GPR99 at the RGC layer of P1 Syrian golden hamsters (S1Q–S1R Fig).

### The Krebs cycle intermediates succinate and α-KG reorganize GC morphology and increase axon growth

**Fig 2:**
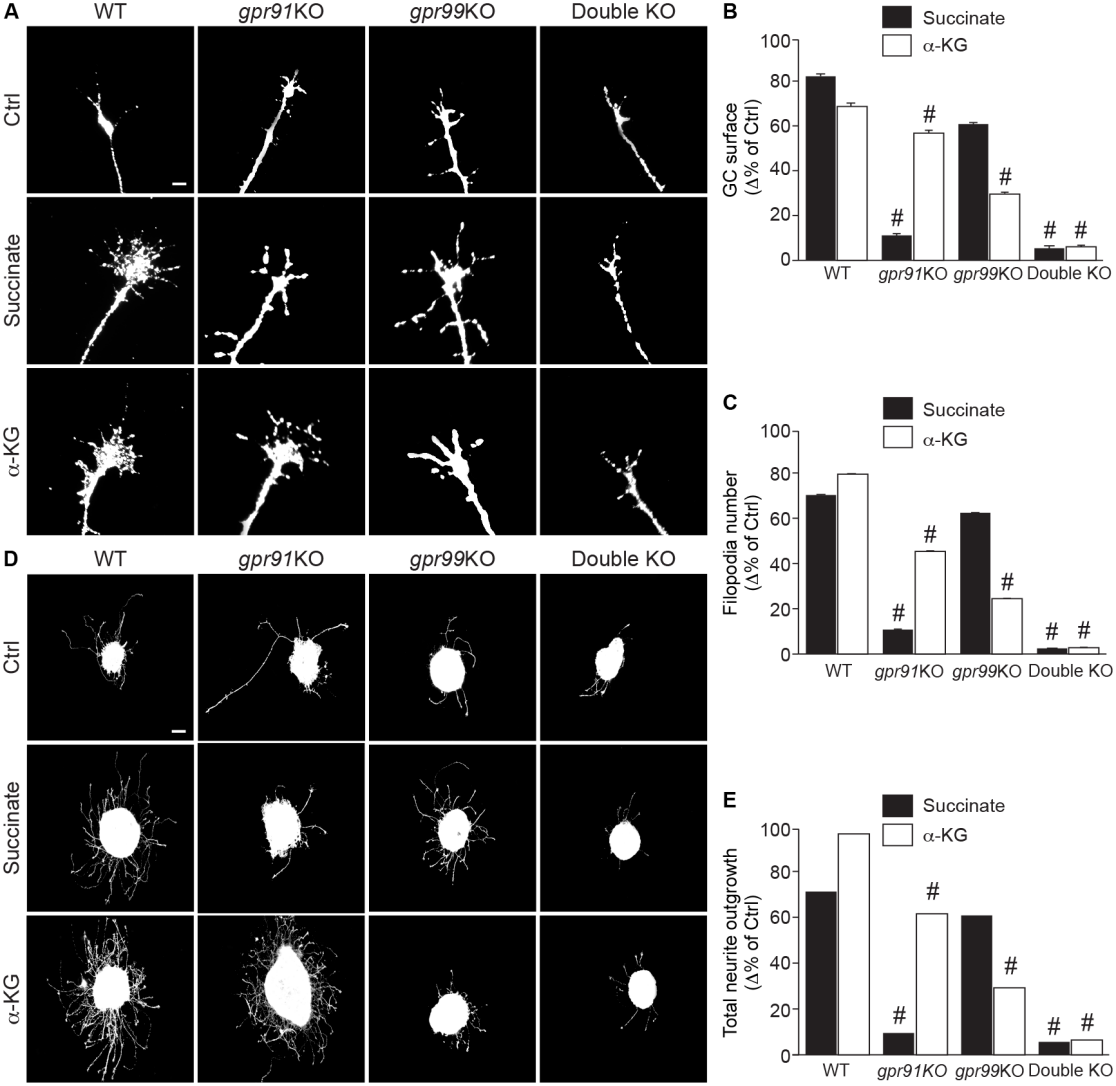
Succinate and α-KG modulate GC morphology and increase neurite outgrowth via GPR91 and GPR99. **(A)** Photomicrographs of E14/15 GCs of retinal projections from WT, *gpr91*KO, *gpr99*KO, and double-KO mouse embryos, untreated and after treatment with GPR91 agonist (100 μM succinate) or GPR99 agonist (200 μM α-KG). **(B)** Analysis of GC surface area (*N* = 104–271 per condition) and **(C)** filopodia number (*N* = 105–271 per condition) following a 1 h treatment with GPR91 or GPR99 ligands in WT, *gpr91*KO, *gpr99*KO, and double-KO mice. **(D)** Photomicrographs of retinal explants from each mouse strain cultured for 1 DIV and treated for 15 h with succinate (100 μM) or α-KG (200 μM). **(E)** Quantification of neurite growth after treatment with GPR91 or GPR99 agonist (*N* = 84–181 per condition). Scale bars: 5 μm **(A)**; 100 μm **(D)**. Values are presented as the means ± SEM. # indicates significant changes compared to WT in **B**, **C**, and **E**; *p* < 0.001. Underlying data can be found in S1 Data. α-KG, α-ketoglutarate; Ctrl, control; DIV, day in vitro; E14/15, embryonic day 14/15; GC, growth cone; KO, knockout; WT, wild-type.

As previous studies have shown that GPCRs are involved in axon guidance, we evaluated the roles of GPR91 and GPR99 on GC actions using retinal explants isolated from E14/15 mouse embryos after 2 days in vitro (DIVs) in culture. Explants treated for 60 min with the specific agonists succinate (100 μM) or α-KG (200 μM) showed a significant increase in the GC surface area and the number of filopodia, compared to controls (Fig 2A–2C and S2A–S2D Fig). As expected, the effect of succinate on GC size and filopodia number was completely abolished in *gpr91*KO but not in *gpr99*KO mouse retinal explants, demonstrating a specific action of succinate on GPR91 (Fig 2A–2C and S2A–S2D Fig). Similarly, α-KG effects were maintained in *gpr91*KO and decreased in *gpr99*KO. The effects of both agonists were abolished in the retinal explants from double-KO *(gpr91*KO*/gpr99*KO) mice (Fig 2A–2C and S2A–S2D Fig). Moreover, following 60-min succinate (100 μM) or α-KG (200 μM) treatment, similar effects were observed on GC surface area and filopodia number of cortical neurons (2 DIVs); these effects were also abolished in neurons lacking the expression of GPR91 and/or GPR99 (S2E–S2G Fig).

To further evaluate the effects of GPR91 and GPR99 ligand treatment on axon growth, retinal explants from WT mouse embryos were treated for 15 h with succinate (100 μM) or α-KG (200 μM). Both agonists induced an increase in total neurite growth (Fig 2D and 2E). Moreover, stimulation of *gpr91*KO murine retinal explants with α-KG and the stimulation of *gpr99*KO murine retinal explants with succinate also induced neurite growth (Fig 2D and 2E). Again, the effects of succinate were essentially abolished in *gpr91*KO murine retinal explants, whereas the increased outgrowth produced by α-KG was markedly reduced in *gpr99*KO murine retinal explants. In double-KO murine retinal explants, the effect produced by either succinate or α-KG was abolished (Fig 2D and 2E).

To investigate whether the effects of intermediates of carbohydrate metabolism on GC morphology and neurite outgrowth could also affect cell viability, we treated murine embryonic retinal explants or cortical neurons with succinate or α-KG and then used a LIVE/DEAD assay to evaluate cell death. Following a 15-h treatment with succinate (100 μM) or α-KG (200 μM), retinal explants or cortical neurons showed no differences in cell viability compared to control explants (S3 Fig). However, we observed a high induction of cell death in the positive control condition of staurosporine-treated explants or neurons (S3 Fig).

Taken together, these results indicate that the Krebs cycle intermediates succinate and α-KG, via GPR91 and GPR99, increase axon growth in retinal explants and modulate GC morphology in retinal explants and primary neurons.

### GPR91 and GPR99 agonists modulate GC morphology and increase axon growth via the extracellular signal–regulated kinases 1 and 2 (ERK_1/2_) pathway

GPR91 is coupled to at least two signaling pathways, G_i_/G_o_ and G_q11_, whereas the activation of GPR99 by α-KG triggers a G_q_-mediated pathway [2]. Moreover, previous reports have demonstrated that succinate activates the mitogen-activated protein kinase (MAPK) signaling pathways via GPR91 [2,12,13,18,19,25]. Since MAPKs mediate axon outgrowth, migration, and guidance [26], we determined whether the effects observed with succinate/GPR91 and α-KG/GPR99 were mediated via the ERK_1/2_ pathway.

**Fig 3:**
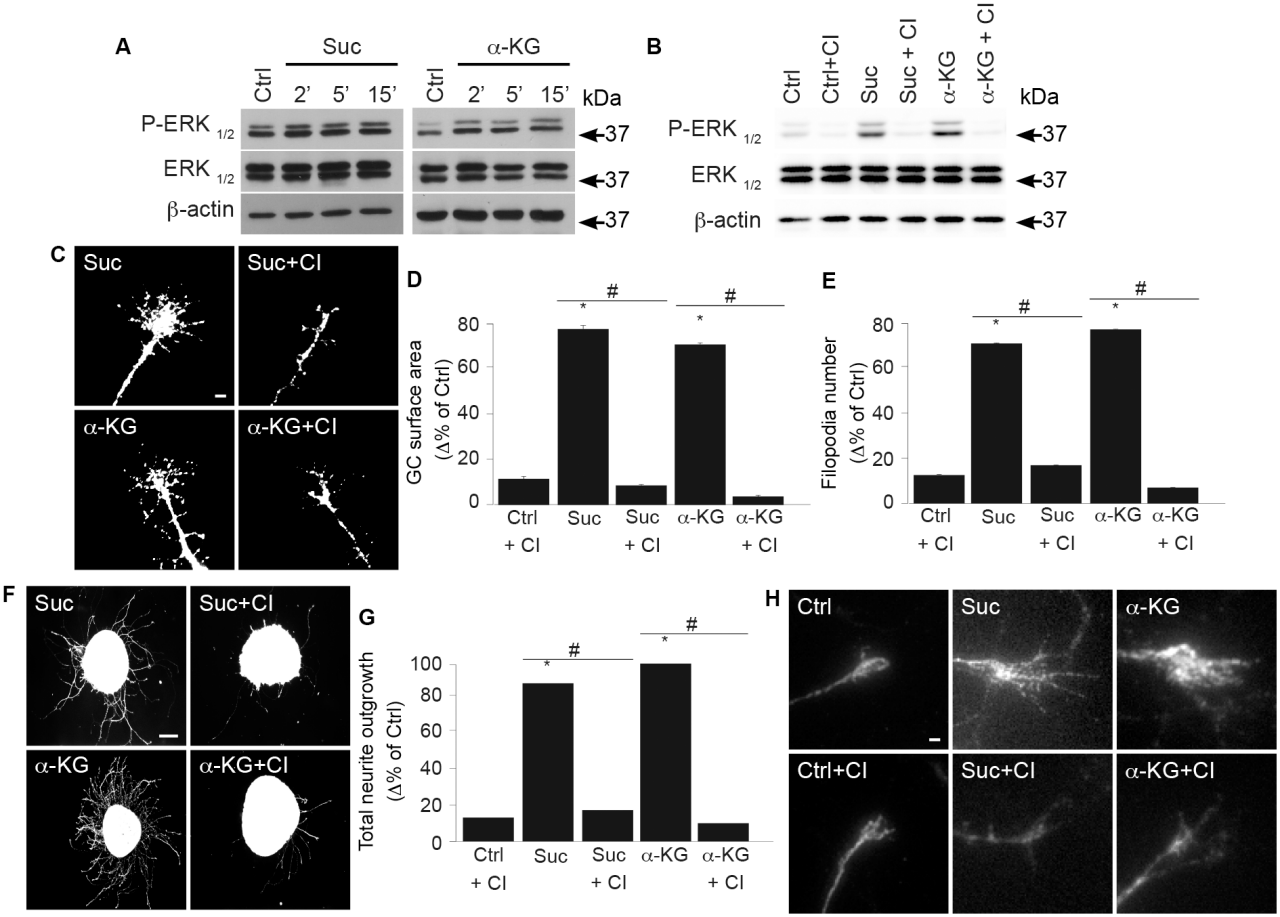
GPR91 and GPR99 agonists modulate GC morphology and increase axon growth via the ERK_1/2_ pathway. **(A)** Protein expression levels of P-ERK_1/2_, ERK_1/2_, and β-actin in primary neuron cultures incubated with one of the following: 100 μM succinate or 200 μM α-KG at 37 °C for 2, 5, and 15 min. The antibody β-actin was used to verify equal loading in all lanes. **(B)** ERK phosphorylation state following 15 min pretreatment with CI-1040, an ERK_1/2_ inhibitor, before incubation with or without GPR91 or GPR99 agonist. **(C)** Photomicrographs of retinal explant GCs treated with GPR91 or GPR99 agonist in the presence or absence of CI-1040. **(D)** Quantification of the GC surface areas (*N* = 178–189 per condition) and **(E)** filopodia numbers (*N* = 178–237 per condition). **(F)** Photomicrographs of retinal explants treated with succinate (100 μM) or α-KG (200 μM) in the presence or absence of CI-1040. **(G)** Analysis of retinal projection growth of retinal explants (*N* = 31–86 per condition). **(H)** Immunohistochemical photomicrographs of the ERK_1/2_ phosphorylation state in retinal explant GCs treated with 100 μM succinate or 200 μM α-KG in the presence or absence of CI-1040. Scale bars: 5 μm **(C, H)**; 100 μm **(F)**. Values are presented as the means ± SEM. * indicates a significant change compared to the Ctrl group in **D, E** and **G**; *p* < 0.0001. # indicates a significant change induced by ERK inhibitor treatment in **D, E** and **G**; p < 0.002. Underlying data can be found in S1 Data. α-KG, α-ketoglutarate; Ctrl, control; ERK_1/2_, extracellular signal–regulated kinases 1 and 2; GC, growth cone.

ERK_1/2_ phosphorylation was significantly increased, both in vitro in neurons and ex vivo in retinal explants, following succinate and α-KG stimulation, while these effects were abrogated by CI-1040, a selective ERK_1/2_ inhibitor (Fig 3A, 3B and 3H). CI-1040 treatment also abolished succinate- and α-KG-induced increases in GC surface area and filopodia number (Fig 3C–3E); no significant differences were observed between the untreated control and a control pretreated with CI-1040. Inhibition of ERK_1/2_ interfered with succinate- and α-KG-induced projection length (Fig 3F and 3G), whereas CI-1040 treatment alone had no significant effect on the total projection length, as observed in control conditions. Moreover, CI-1040 treatment did not affect the viability of embryonic retinal explants and cortical neurons, as no significant neuronal cell death was observed compared to controls with the LIVE/DEAD assay (S3 Fig). These data implicate the ERK_1/2_ pathway in the GPR91- and GPR99-induced modulation of GC morphology and axon outgrowth via their respective TCA cycle metabolite ligand.

### Role of GPR91 and GPR99 during development

To determine the contribution of GPR91 and GPR99 to the development of retinal projections in vivo, E14/15 murine embryos received an intraocular injection of DiI (DiIC18[3] [1,1’-dioctadecyl-3,3,3’,3’-tetramethylindocarbocyanine perchlorate]), a lipophilic tracer. After 7 d of tracer diffusion, surgery was performed to visualize the optic nerve, chiasm, and tract. The photomicrographs obtained revealed that genetic deletion of either GPR91 or GPR99 had no detrimental effects on RGC axon guidance, as axon steering at the optic chiasm, after a single genetic deletion of *gpr91* or *gpr99*, was similar to the WT group (S4A and S4B Fig). Moreover, succinate and α-KG treatment also failed to modulate axon steering in time-lapse microscopy experiments performed on GCs from E14/15 WT murine retinal explants at 1 DIV (S5A–S5E Fig). Microgradient application of succinate or α-KG did not induce any significant directional GC turning compared to the vehicle control (S5A–S5E Fig). Interestingly, short-term exposure to succinate induced an increase in the growth of retinal axons, while α-KG exposure had no significant effects (S5E Fig). However, in double-KO mice, few retinal axon fibers projected to the ipsilateral side of the brain although, some extended into the contralateral optic nerve. The concomitant absence of GPR91 and GPR99 appeared to induce some abnormal projections in the ipsilateral and contralateral sides of the optic chiasm, suggesting a potential compensatory role played by each receptor in the absence of the other (S4A and S4B Fig). Moreover, to assess the involvement of the citric acid cycle intermediate receptors in retino-geniculate development, we examined the projections to the dorsal lateral geniculate nucleus (dLGN) of adult mice. Contralateral and ipsilateral projections in the dLGN from all genetically modified mouse strains occupied the same area as those of WT mice (S4C Fig). These data indicate a similar overlap between contralateral and ipsilateral RGC projections in the dLGN for all mouse genotypes (S4D Fig). Taken together, these observations demonstrate that GPR91 and GPR99 do not appear to be implicated in guidance and target selection during the development of the retinogeniculate pathway in vivo.

**Fig 4:**
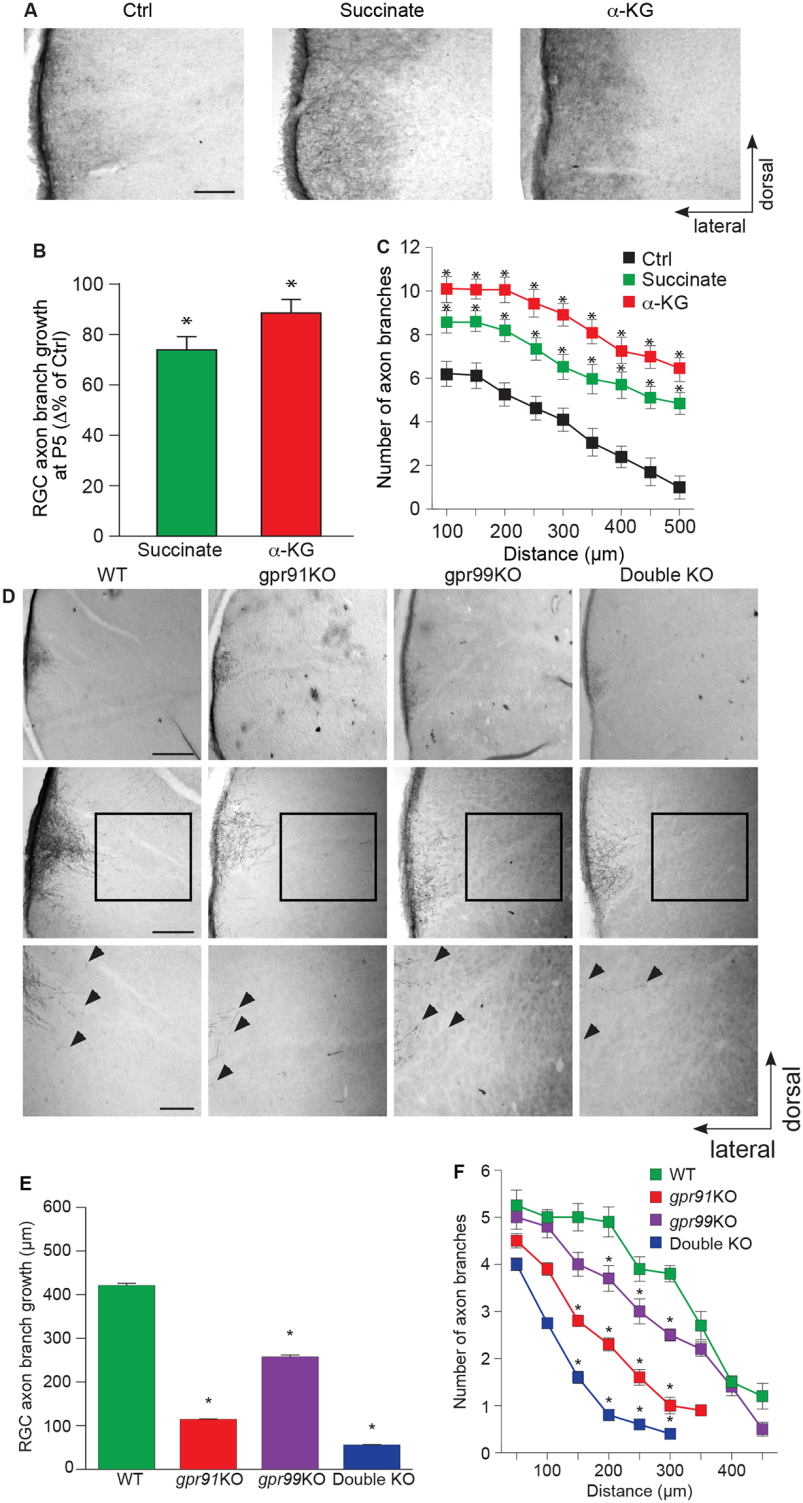
Succinate/GPR91 and α-KG/GPR99 are involved in RGC axon growth in vivo. **(A)** Photomicrographs of P5 hamster retinal projections in the DTN for the control, succinate (100 mM), and α-KG (200 mM) treatment groups. **(B, C)** Quantification of retinal projection development in the DTN: collateral axon projection length (*N* = 168–240 per condition) and collateral axon branch number (*N* = 34–57 per condition) of treated groups compared with the Ctrl group. Succinate and α-KG treatment increased axon growth and collateral branch number compared with the Ctrl. **(D)** Photomicrographs of P5 retinal projections in the DTN in WT, *gpr91*KO, *gpr99*KO, and double-KO mice. **(E)** Quantification of retinal projections in the DTN during the development of WT, *gpr91*KO, *gpr99*KO, and double-KO mice (*N* = 627–963 per condition). Collateral projection length is expressed as the mean ± SEM. **(F)** The number of collateral axon branches decreased in *gpr91*KO, *gpr99*KO, and double-KO mice compared to WT (*N* = 82–160 per condition). Data are presented as the means ± SEM. Scale bars: 100 μm **(A, D)**. * indicates a significant change compared to the Ctrl group in **B** and **C** (*p* < 0.05) and in **E** and **F** (*p* < 0.0001). Underlying data can be found in S1 Data. α-KG, α-ketoglutarate; Ctrl, control; DTN, dorsal terminal nucleus; KO, knockout; RGC, retinal ganglion cell; WT, wild-type.

To investigate the in vivo effects of intermediates of carbohydrate metabolism during the development of the visual system, the mouse model presents limitations. Because the mouse visual system is completed at birth [27], we further utilized a different rodent model. The Syrian golden hamster has a shorter gestation period (15 d versus 18.5 d), and pups are born with a relatively premature visual system [27]. As the axons of RGCs reach their thalamic and midbrain targets at P3 in the hamster, this model allows examination of the induction of axon growth by different agonists [10,28]. Taking advantage of this observation, hamsters were injected intravitreally 24 h after birth (P1) with a mixed solution of cholera toxin subunit B (CTb) with either 0.9% saline solution, 100 mM succinate, or 200 mM α-KG, and immunohistological analyses were performed at P5. Intraocular injections of CTb produced intense labeling of thalamic and midbrain targets such as the dLGN and superior colliculus, making the evaluation of the collateral growth of RGC axons difficult. Thus, we evaluated the RGC branch growth at the dorsal terminal nucleus (DTN), one of the nuclei composing the accessory visual pathway and involved in mediating visuomotor reflexes underlying the generation of optokinetic nystagmus [29]. Compared with the control group, unilateral intraocular injections of succinate or α-KG induced significant increases in RGC collateral axon projection length and branch number in the DTN (Fig 4A–4C).

We next proceeded to investigate the impact of genetic deletions of *gpr91* and *gpr99* on axon growth during development in vivo. Within 24 h of birth, pups from all 4 murine genotypes received a unilateral intraocular injection of CTb to label their retinal projections. At P5, immunohistological experiments revealed the effects of GPR91 and GPR99 on RGC axon development. Investigating RGC branch growth at the DTN, we showed a significant decrease in the collateral projection lengths of the KO animals compared to the control group (Fig 4D and 4E). In addition, axon collateral density was significantly decreased in *gpr91*KO, *gpr99*KO, and, to a greater extent, in double-KO mice, compared to WT controls (Fig 4F). These findings demonstrate—for the first time, to our knowledge—the essential role of GPR91 and GPR99 in the growth of RGC projections.

## Discussion

Most functional studies of GPR91 and GPR99, receptors of intermediates of carbohydrate metabolism, have been performed outside the central nervous system, primarily in the kidney and heart [2,14,15]. In the present study, we showed that GPR91 and GPR99 are expressed on axonal and dendritic projections, GCs and filopodia of murine embryonic retinal explants, and on retinal projections and cell body of RGCs during the development of the retinothalamic pathway. We demonstrated that succinate and α-KG increase ERK_1/2_ phosphorylation, corroborating a large number of studies on signaling pathways triggered by GPR91 [2,12,18,25]. Moreover, stimulation of both GPR91 and GPR99 resulted in the modulation of GC morphology and an increase in RGC axon growth in an ERK_1/2_-dependent manner. The increased GC size, number of filopodia, and growth of RGC axons following stimulation of GPR91 and GPR99 by succinate and α-KG, respectively, is the first report, to our knowledge, implicating these ligands and receptors in axon growth. Interestingly, the deletion of GPR91 completely blocked the effects of succinate but also partially abolished the effects observed with α-KG. Nevertheless, in double-KO animals, the effects of both succinate and α-KG were abrogated. These results tend to demonstrate that succinate’s effects on RGC axon growth were mediated only through GPR91, while α-KG could, through an as-yet-unknown mechanism, activate both GPR91 and GPR99. A possible mechanism could be the conversion of α-KG into succinate, since α-KG is a precursor of succinate in the Krebs cycle. Moreover, our findings showed that GPR91 and GPR99, while having no effect on axon guidance, have complementary roles in RGC axon growth during development.

These data are consistent with previous observations in which succinate, via GPR91, has shown highly proliferative and stimulating vascular effects in different tissues [16,17], to promote chemotaxis [19,30] and to potentiate the activation and aggregation of platelets [18,31]. Axon guidance and angiogenesis share several fundamental challenges during the formation of their extensive networks. Tip cells—specialized endothelial cells at the end of each vessel sprout—are motile and dynamically extend long filopodia protrusions reminiscent of axonal GCs [32]. In light of the spatiotemporal link between axon growth and angiogenesis, as well as the morphological similarities between endothelial tip cells and axonal GCs, the observed increase in the morphology of GC and neurite growth could be explained by a similar mechanism in the presence of succinate.

As the only type of neuron that sends axons out of the retina, RGCs ensure the visual and cognitive processing of information from the outside world to the brain. A combination of intrinsic and extrinsic signals also plays an important role in driving the axons through the visual pathway via responsive GCs, which detect and effectively translate a multitude of external chemotactic cues. In the mouse, the axon decussation occurs at the level of the optic chiasm at around E14–16 [33]. We observed that in WT, *gpr91*KO, or *gpr99*KO mice, the optic chiasm appeared relatively normal, as the majority of the axons at the midline crossed to project contralaterally. Our results suggest that in the mouse visual system, the absence of either GPR91 or GPR99 is insufficient to affect decussation. Moreover, neither GPR91 nor GPR99 activity at the GC modulated axon turning in an ex vivo experiment of retinal explants, since GCs are not attracted nor repelled in the presence of a succinate or α-KG microgradient, whereas succinate induced significant axon extension. Based on these results, succinate plays an essential role in axon growth by increasing axon motility, but succinate and α-KG do not affect GC and axon guidance. However, the visual projections of double-KO mice showed some mild abnormalities in axon guidance that could be explained by a compensatory effect between the two receptors, which would allow a rescue of this mild phenotype in *gpr91*KO or *gpr99*KO mice. Nevertheless, further experiments are needed to study this subtle defect in a more quantitative fashion in order to draw significant conclusions. In addition, our data show that deletion of either GPR91 or GPR99 in vivo did not affect target selection of retinal projections. Indeed, during perinatal development, RGC axons connect with multiple targets in the dLGN, sharing common terminal space, while RGC axons occupy distinct eye-dependent nonoverlapping regions of the dLGN in the adult rodent. Eye-specific segregation only occurs during postnatal development [34]. Accordingly, a similar relative eye-specific segregation of retinal projections was observed in the adults of all 4 mouse genotypes. Thus, our in vivo results support previous ex vivo findings that GPR91 and GPR99 do not modulate RGC axon guidance and target selection during the establishment of the visual pathway.

However, we demonstrated that TCA cycle intermediates induce axon growth in vivo during the development of the visual system, as intraocular injection of succinate and α-KG induced significant increases in RGC collateral axon projection length and branch number in the DTN. Moreover, accordingly, genetic interference with GPR91 or GPR99 activity profoundly affects retinal projection growth in the DTN. We showed a significant difference between WT, *gpr91*KO, and *gpr99*KO mice in axon projection length and branching at the DTN. Furthermore, the relative lack of growth of retinal projections in double-KO mice demonstrates the fundamental role played by GPR91 and GPR99 during RGC axon growth. Nonetheless, these in vivo experiments do not conclude that the receptors involved in the growth-promoting actions of intermediates of carbohydrate metabolism are only those expressed at the GCs but could also be, to some extent, those expressed throughout the projections or on the cell body of RGCs as well.

The levels of intermediates of carbohydrate metabolism adapt depending on tissue needs and the conditions in the surrounding regions. Investigating RGC projections and GC actions in the developing visual system faces technical limitations regarding intermediates of carbohydrate metabolism dosing. The amount of tissue needed (and its isolation) from mouse embryos or hamster newborn pups does not allow detection of metabolites due to the technique sensitivity and the rapid turnover of the metabolites. Nevertheless, based on previous published data and our own findings, we sought to avoid nonspecific responses by determining the lowest responsive doses for succinate and α-KG in our system, even if the physiological levels could not be measured [2,3,16–18].

In summary, this study demonstrates—for the first time, to our knowledge—a role for the intermediates of carbohydrate metabolism succinate and α-KG and their respective receptor GPR91 and GPR99 in axon growth during development in vivo. These receptors mediate axon growth in an ERK_1/2_-dependent manner, although succinate and α-KG have no effect on axon guidance. Moreover, these findings suggest a potential link between mitochondria and axon growth in development, outside the strict production of energy. This study not only demonstrates a new role for TCA cycle intermediates in the visual system development but also provides a foundation for the investigation of metabolite receptors in the visual, central, and peripheral nervous system development. This novel concept also provides new avenues for the elaboration of effective therapies aimed at the development and regeneration of the nervous system.

## Materials and methods

### Ethics statement

All experimental procedures were approved by the Animal Care Committee of Sainte-Justine’s Hospital Research Center or the relevant University of Montreal animal care committee’s regulations and were conducted in accordance with the Association for Research in Vision and Ophthalmology statement regarding the use of animals in ophthalmic and vision research and the guidelines established by the Canadian Council on Animal Care.

### Animals

The C57BL/6 WT control mice were purchased from Jackson Laboratory. Syrian golden hamsters (Charles River Laboratories, Saint-Constant, Canada) were used in this study.

### Generation of *gpr91*KO mice

*Sucnr1*KO mice, generated by Deltagen through partial replacement of exon 2 (5’-GGCTACCTCTTCTGCAT-3’) with a lacZ-neomycin cassette, were generously provided by Dr. José M. Carbadillo at Norvartis Institutes for Biomedical Research, Vienna, Austria [19]. As described by Rubic and colleagues in 2008, correctly targeted 129/OlaHsd embryonic stem cells were used for the generation of chimeric mice, which were crossed with C57BL/6 (called “WT” here). F1 mice with germline transmission of the mutated gene were further backcrossed with WT mice for 10 generations (in specific pathogen-free conditions at the Novartis Institutes for Biomedical Research, Vienna) before being intercrossed to produce homozygous *gpr91*KO mice. *gpr91*KO mice were healthy and bred normally when maintained in specific pathogen-free conditions. All experiments in the production of the *gpr91*KO mice were conducted in accordance with Austrian Law on Animal Experimentation and the Novartis Animal Welfare Policy. All procedures were approved by the local government and the animal care and user committee of the Novartis Institutes for Biomedical Research, Vienna.

### Generation of *gpr99*KO mice

Heterozygous (*GPR99*+/−) mice with mixed genetic background (C57BL/6J− Tyrc-Brd x 129 Sv/EvBrd) were developed and generously provided by Lexicon Pharmaceuticals Incorporated (The Woodlands, TX). The full-length *gpr99* gene was removed by homologous recombination as the PCR-generated selection cassette was introduced in a murine genomic clone by yeast recombination, followed by the electroporation of the linearized targeting vector in 129 Sv/EvBrd embryonic stem cells. In selected clones, *gpr99* deletion was confirmed by Southern hybridization followed by their injection into C57BL/6J-Tyrc-Brd blastocysts. To generate F1 heterozygous offspring, the resulting chimeras were backcrossed to C57BL/6J-Tyrc-Brd. Heterozygous mice were intercrossed to generate WT control (*gpr99*WT), homozygous-null (*gpr99*KO), and heterozygous littermates, consistent with Mendelian ratios. The resulting homozygous-null *gpr99*KO mice were backcrossed onto the C57BL/6 background with C57BL/6 obtained from Jackson Laboratory (Connecticut, USA) for 10 generations in CHU Sainte-Justine’s Research Center animal facility before using them in experiments. The *gpr99*KO mice were viable, healthy, and bred normally when maintained in specific pathogen-free conditions.

### Generation of *gpr91*KO / *gpr99*KO double-KO mice

*gpr99*KO / *gpr91*KO mice (double KO) were generated by crossing *gpr99*KO and *gpr91*KO mice to produce *gpr99*+/− / *gpr91*+/− (double-heterozygous) parents. The double-heterozygous parents were then crossed together until we obtained double-KO *gpr99*KO / *gpr91*KO (1:16 pups according to Punnett Square) male and female mice that were then crossed together to obtain a stable double-KO mouse lineage. The double-KO mice were viable, healthy, and bred normally when maintained in specific pathogen-free conditions.

### Genotypic screening

Mice were genotyped by PCR reactions of tail genomic DNA using specific primers for either the WT or mutant allele.

For GPR91 mice, the primer pair WT-F: 5′-GTTCATTTTTGGACTGCTTGGG-3′ and WT-R: 5′-AATGGCAAATTCCTTCTTTTGTAGA-3′ generated a GPR91-specific fragment only present in the WT allele, while the primer pair KO-F: 5′- GGCACATATCGGTTGCTTATACAGA-3′ and KO-R: 5′- GGGTGGGATTAGATAAATGCCTGCTCT-3′ amplified a fragment specific to the selection cassette of the *gpr91*KO mutant allele. For GPR99 mice, a GPR99-specific fragment present in the WT but absent in the mutant allele was generated using the specific primer pair UTT069-21 (5′-GAGCCATGATTGAGCCACTG-3′) and UTT069-25 (5′-CACCACTGGCATAGTAATGG-3′). Another primer pair amplified a fragment specific to the selection cassette of the *gpr99*KO mutant allele: UTT069-3 (5′-CAGAGCCATGCCTACGAG-3′) and GT (5′-CCCTAGGAATGCTCGTCAAGA-3′). For double-KO mice, all pairs of primers were used (4 reactions) to determine whether both genetic modifications were present.

### Reagents

BSA, ciliary neurotrophic factor, DNase, forskolin, Hoechst 33258, insulin, laminin, poly-D-lysine, progesterone, selenium, putrescine, succinate, α-KG, trypsin, and triiodothyronine were purchased from Sigma Aldrich (Oakville, ON, Canada). B27, N2, Dulbecco’s phosphate-buffered saline, FBS, glutamine, Neurobasal medium, penicillin-streptomycin, Minimum Essential Medium Eagle Spinner Modification (S-MEM), and sodium pyruvate were purchased from Life Technologies (Burlington, ON, Canada). The standard donkey and goat sera were from Jackson ImmunoResearch (West Grove, PA, USA). ERK_1/2_ inhibitor (CI-1040) was obtained from Selleck Chemicals (Houston, TX, USA). LNAC was acquired from EMD (La Jolla, CA, USA). The CTb was from List Biological Laboratories (Campbell, CA, USA). Triton X-100 was purchased from US Biological Life Sciences (Salem, MA, USA). DiI stain was obtained from Molecular probes (Eugene, OR, USA).

### Tissue preparation for immunohistochemistry

Adult mice and P1 hamsters were euthanized by an overdose of isoflurane. Transcardiac perfusion was conducted with phosphate-buffered 0.9% saline (PBS; 0.1 M, pH 7.4), followed by 4% formaldehyde in PBS, until the head was fixed. The nasal part of the eyes of murine embryos and adult mice was marked with a suture and removed. Two small holes were made in the cornea before a first postfixation step in formaldehyde for a period of 30 min. The cornea and lens were removed, and the eyecups were postfixed for 30 min in formaldehyde. The eyecups were then washed in PBS, cryoprotected in 30% sucrose overnight, embedded in NEG 50 tissue Embedding Media (Thermo Fisher Scientific Burlington, ON, Canada), flash-frozen, and kept at −80 °C. Sections (14-μm thick) were cut with a cryostat (Leica Microsystems, Concord, ON, Canada) and placed on gelatin/chromium-coated slides.

### Immunohistochemistry

Retinal sections were washed in 0.1 M PBS, postfixed for 5 min in a 70% solution of ethanol, rinsed in 0.03% Triton X-100 in PBS, and blocked in 10% normal donkey serum and 0.5% Triton X-100 in PBS for 1 h. The sections were then incubated overnight with antibodies against GPR91 or GPR99. The antibody Brn-3a was also used as a specific marker for RGCs. After incubation with the primary antibodies, the sections were washed in PBS, blocked for 30 min, and incubated for 1 h with the secondary antibodies Alexa Fluor 647 donkey anti-rabbit and Alexa Fluor 488 donkey anti-mouse. After washing, the sections were mounted using a homemade PVA-Dabco medium.

### Antibody characterization

The specifications of all the antibodies used in this study are detailed in S1 Table.

### Confocal microscopy

Images of the central retina (within 200 μm of the optic nerve head) were taken using a laser scanning confocal microscope (TCS SP2, Leica Microsystems) with a 40X (NA: 1.25) oil immersion objective and 488 and 633 nm lasers. Image stacks (1,024 × 1,024 pixels × 0.5 μm per stack) were captured with a frame average of 3 using the LCS software (version 2.6.1; Leica Microsystems). The stacks were taken sequentially and in distant wavelengths to ensure no “bleed through” between channels and were collapsed into projection images. All images in which labeling intensities were compared were obtained under identical conditions of gain intensity. Because gray-scale photographs provide better contrast and more detail, individual channels are presented in gray scale, and the merged images are presented in color.

### Retinal explant culture

The retinas were isolated from E14/15 mouse embryos, dissected into small segments in ice-cold Dulbecco’s phosphate-buffered saline, and plated on 12-mm glass coverslips previously coated with poly-D-Lysine (20 μg/ml) and laminin (5 μg/ml) in 24-well plates. The explants were cultured in Neurobasal supplemented with 100 U/ml penicillin, 100 μg/ml streptomycin, 5 μg/ml LNAC, 1% B27, 40 ng/ml selenium, 16 μg/ml putrescine, 0.04 ng/ml triiodo-thyronine, 100 μg/ml transferrin, 60 ng/ml progesterone, 100 μg/ml BSA, 1 mM sodium pyruvate, 2 mM glutamine, 10 ng/ml ciliary neurotrophic factor, 5 μg/ml insulin, and 10 μM forskolin at 37 °C and 5% CO_2_. At 0 DIV, 1 h following plating, the explants were treated for 15 h for projection analysis or for 1 h at 1 DIV for GC analysis. The photomicrographs were taken using an Olympus IX71 microscope (Olympus, Markham, ON, Canada) and analyzed with Image-Pro Plus 5.1 software (Media Cybernetics, Bethesda, MD, USA). The total length of axon bundles was quantified and expressed as the mean ± SEM. Statistical significance of differences between means was evaluated by analysis of variance (ANOVA) with Bonferroni’s post-hoc test (Systat Software Inc, Chicago, IL, USA).

### Primary neuron culture

Primary cortical neurons were used in this study because of the large number of neurons that can easily be cultured and harvested for biochemical assays, which is hardly possible with RGCs. C57BL/6 WT, *gpr91*KO, *gpr99*KO, and double-KO pregnant mice were used. Brains from E14/15 embryos were dissected, and the superior layer of each cortex was isolated and transferred in 2 ml S-MEM containing 2.5% trypsin and 2 mg/ml DNase and incubated at 37 °C for 15 min. The pellet was transferred into 10 ml S-MEM with 10% FBS and stored at 4 °C. After centrifugation, the pellet was again transferred in 2 ml S-MEM supplemented with 10% FBS and triturated 3 to 4 times. The supernatant was transferred in 10 ml Neurobasal medium. Dissociated neurons were counted and plated at 50,000 cells per well on 12 mm glass coverslips previously coated with poly-D-lysine (20 μg/ml) for immunocytochemistry or at 250,000 cells per 35 mm petri dish for western blot. Neurons were cultured for 2 d in Neurobasal medium supplemented with 1% B-27, 100 U/ml penicillin, 100 μg/ml streptomycin, 0.25% N2, and 0.5 mM glutamine. They were then treated with either a GPR91 agonist (100 μM succinate), GPR99 agonist (200 μM αKG), or ERK_1/2_ inhibitor (20 μM CI-1040) for 1 h to study GC morphology or 2, 5, and 15 min for ERK_1/2_ quantification using western blot analysis.

### Determination of cell viability

LIVE/DEAD cell viability assay: Cell viability was assessed with the LIVE/DEAD assay using an ethidium homodimer/calcein acetoxy methyl ester (L-3224, Molecular Probes, Eugene, OR, USA) combination of vital dyes, as previously described [35,36]. Staurosporine (5 μM), an inducer of apoptotic cell death, was used as a positive control [37].

### Immunocytochemistry

After treatment, retinal explants and primary cortical neuron cultures were washed with PBS (pH 7.4), fixed in 4% formaldehyde (pH 7.4), and blocked with 2% normal goat serum (NGS) and 2% BSA in PBS containing 0.1% Tween 20 (pH 7.4) for 30 min at room temperature. The samples were then incubated overnight at 4 °C in a blocking solution containing anti-GAP-43, anti-GPR91, anti-GPR99, anti-MAP2, or anti-NFM. The following day, the samples were washed and labeled with Alexa Fluor 488 and 555 secondary antibodies and Hoechst 33258 (1:10,000), and the coverslips were mounted with a homemade PVA-Dabco medium [38].

### Western blot analysis

Primary cortical neurons were cultured for 2 DIVs at a density of approximately 250,000 cells/dish in 35 mm poly-D-lysine-coated petri dishes. Following treatment, neurons were washed once with ice-cold PBS (pH 7.4) and then lysed with Laemmli sample buffer. Thirty micrograms of protein/sample of the homogenate were resolved with 12% SDS-polyacrylamide gel electrophoresis, transferred onto a nitrocellulose membrane, blocked with 5% BSA, and incubated overnight with antibodies directed against ERK_1/2_, p-ERK_1/2_, and β-actin, the latter serving as a loading control. The blots were exposed to the appropriate HRP-coupled secondary antibodies (Jackson Immunoresearch Laboratories, West Grove, PA, USA). Detection was performed using homemade enhanced chemiluminescence western blotting detection reagent (final concentrations: 2.5 mM luminol, 0.4 mM p-coumaric acid, 0.1 M Tris-HCl [pH 8.5], 0.018% H_2_O_2_).

### GC behavior assay

Embryonic retinal explants were cultured on a coverglass in a borosilicate chamber (Lab-Tek; Rochester, NY, USA) for 2 DIVs and placed in an incubator mounted on an inverted microscope (Olympus IX71). They were maintained at 37 °C and 5% CO_2_ with a live cell chamber (Neve Bioscience, Camp Hill, PA, USA) throughout the whole experiment. A microgradient was created using a Picoplus micro-injector (Harvard Apparatus, St-Laurent, QC, Canada). Glass micropipettes with a tip of 2–3 μm diameter were positioned at 45° and at 100 μm away from the GC of interest, as described previously [8,10,11].

### Intraocular injections

Syrian golden hamsters (Charles River) were used for investigating the in vivo implication of succinate/GPR91 and α-KG/GPR99 in RGC projection growth during postnatal development. At P1, 24 h after birth, anesthetized hamsters received a unilateral injection of 2 μl solution of CTb with either 0.9% saline solution, succinate (100 mM), or α-KG (200 mM). Briefly, under an operating microscope, a small incision was made in the eyelids to access the right eye. The injections were administered using a glass micropipette attached to a 10 μl Hamilton syringe. The micropipette was carefully inserted into the vitreous at an angle to avoid damage to the lens. Following the injection, the eyelids were closed with surgical glue (Vetbond; 3M).

At P5, 4 d after the injection, hamsters were anesthetized and perfused transcardially with 0.1 M PBS, pH 7.4, followed by 4% PFA in PBS. The brains were removed, postfixed overnight at 4 °C and cryoprotected with sucrose. Then, brains were frozen and kept at −80 °C until processing by immunohistochemistry according to a protocol previously described by Argaw and colleagues in 2011 [8]. Briefly, 40 μm—thick coronal sections of tissue were incubated in 90% methanol and 0.3% H_2_O_2_ in 0.1 m PBS, pH 7.4, for 20 min. They were then rinsed and incubated in 0.1 M glycine/PBS for 30 min, followed by an overnight incubation (4 °C) in PBS containing 4% NDS, 2.5% BSA, and 1% Triton X-100. The sections were subsequently rinsed and immersed for 48 h at room temperature in a solution containing goat anti-CTb diluted 1:4,000 in PBS with 2% NDS, 2.5% BSA, and 2% Triton X-100. Afterward, the sections were rinsed and incubated in 2% NDS and 2.5% BSA/PBS for 10 min. This was followed by a 1 h incubation in donkey anti-goat biotinylated secondary antibody diluted 1:200 in PBS with 2% NDS, 2.5% BSA, and 1% Triton X-100. Tissues were rinsed, incubated in 2% NDS and 2.5% BSA in PBS for 10 min, and subsequently processed with an avidin-biotin-peroxidase complex ABC Kit (diluted 1:100 in PBS) for 1 h in the dark at room temperature. The sections were then rinsed and preincubated in 3, 3′-diaminobenzidine tetrahydrochloride (DAB) in PBS for 5 min. The peroxidase reaction product was visualized by adding 0.004% H_2_O_2_ to the DAB solution for 2–4 min. Sections were finally washed 5 times (1 min each) with PBS, mounted on gelatin-chromium alum-subbed slides, air-dried, dehydrated in ethanol, cleared in xylenes, and mounted on coverslips with Depex (EMS).

### Lipophilic dye tracing and tissue clearing

After 14–15 d of gestation, pregnant mice (WT, *gpr91*KO, *gpr99*KO, and double KO) were euthanized, and the embryos were removed. The lambdoid sutures of the embryos were incised, and the occipital bones were removed to expose the brain to the fixative (4% formaldehyde), where they were placed for 1 wk at 4 °C until tracing with DiI. For complete optic nerve labeling, 1 eye of each embryo was enucleated and crystals of DiI implanted unilaterally into the optic disk. Embryos were incubated at 37 °C in 4% formaldehyde for 7 d. Tissue clearing was performed according to Hama and colleagues (2011) [39]. Briefly, embryos were incubated for 2 d in Scale A2 solution (4 M urea, 10% glycerol, 0.1% Triton X-100, in water) followed by 2 d in Scale B4 solution (8 M urea, 0.1% Triton X-100, in water) and then to a fresh Scale A2 solution for 1 wk to complete the clearing [39]. The brains were then carefully removed with their optic nerves, and the proximal visual system was imaged with a fluorescence microscope to allow the observation of subtle guidance defects at the optic chiasm.

### Tracing of RGC axons

For eye-specific segregation studies in the dLGN, C57BL/6 WT, *gpr91*KO, *gpr99*KO, and double-KO adult mice received an intraocular injection of CTb conjugated to Alexa Fluor 555 into the left eye and CTb coupled to Alexa Fluor 488 into the right eye (2 μl; 0.5% in sterile saline). Four days after the injection, the animals were anesthetized and perfused transcardially with 0.1 M PBS (pH 7.4) followed by 4% formaldehyde. The brains were removed, postfixed overnight at 4 °C, cryoprotected, frozen, and kept at −80 °C. Retinal projections marked with the CTb were visualized on brain sections washed 5 times (1 min each) with PBS, mounted on gelatin-chromium alum-subbed slides, air-dried, and mounted on coverslips with DEPEX (EMS, Hatfield, PA, USA).

### In vivo quantification method

The photomicrographs of the optic chiasm were taken with an IX71 microscope (Olympus, Richmond Hill, ON, Canada), an Evolution VF camera (Media Cybernetics, Warrendale, PA, USA) and Image-Pro Plus 5.1 image analysis software. Universal gains and exposures were established for each labeling. Raw images of the dLGN were imported to MATLAB (Natick, MA, USA), and an area of interest comprising the dLGN was cropped, excluding the ventral lateral geniculate nucleus and the intergeniculate leaflet. Then, the degree of left and right eye projection overlap was quantified using an established multithreshold method of analysis [40–42]. This approach allows for a better analysis of overlapping regions independent of the threshold. For these experiments, an observer “blind” to the experimental conditions to avoid any bias performed the quantification. Values are expressed as the means ± SEM. The significance of differences between means was evaluated by Student *t* test analysis (Systat).

To assess axon growth in vivo, photomicrographs of the DTN of mice and P5 hamsters were taken with a microscope (Leica Microsystems, Concord, ON, Canada) coupled to an Evolution VF camera (Media Cybernetics). The images were quantified using Image-Pro Plus 5.1 software. The growth of axon branches was quantified on consecutive photomicrographs of coronal slices of brain tissue comprising the DTN. On each photomicrograph, the distance between the lateral border of the DTN and the tips of the longest axon branches was measured. To take into account brain size differences, axon branch lengths were normalized with the interthalamic distance (distance between the right and left lateral borders of the thalamus; see S6A Fig for a schematic representation of such quantification). Axon collateral number was quantified on consecutive photomicrographs comprising the DTN using an adaptation of the Sholl technique [43], as described by Duff and colleagues in 2013 [11] and illustrated in S6B Fig. Values are expressed as the means ± SEM.

### Statistical analysis

The significance of differences between means was evaluated by ANOVA with Bonferroni’s post-hoc test (Systat).

## Supporting information

S1 Data Underlying data for Figs 2, 3 and 4, S2, S4 and S5 Figs. (XLSX)

S1 Fig Specificity of the antibodies against GPR91 and GPR99 in the retina. **(A)** Expression of GPR91 protein in the WT adult mouse retina with lack of fluorescence in the *gpr91*KO mouse **(C)**. **(B & D)** GPR99 immunoreactivity in the retina of adult WT and *gpr99*KO mice. **(E & G)** Expression of GPR91 and **(F & H)** GPR99 in retinal sections of E14/15 WT, *gpr91*KO, and *gpr99*KO murine embryos. Scale bars: 75 μm **(A-H)**. Expression of GPR91 and GPR99 in retinal explants, GCs, and neurites of E14/15 WT **(I-L)**, *gpr91*KO, and *gpr99*KO murine embryos **(M-P)**. Scale bars: 100 μm **(I & K)**; 10 μm **(J & L)**. Expression of GPR91 and GPR99 proteins in retinal sections of P1 hamster pups **(Q, R)**. Scale bars: 10 μm. E14/15, embryonic day 14/15; GC, growth cone; GCFL, ganglion cell fiber layer; GCL, ganglion cell layer; KO, knockout; NBL, neuroblast layer; WT, wild-type.
(TIF)

S2 Fig Succinate and α-KG modulate GC morphology and filopodia number of retinal explants and cortical neurons via GPR91 and GPR99. **(A-D)** Additional representative examples of E14/15 GCs of RGC projections from WT, *gpr91*KO, *gpr99*KO, and double-KO mouse embryos **(E)** Photomicrographs of E14/15 GCs of cortical neurons from WT, *gpr91*KO, *gpr99*KO, and double-KO mouse embryos after a 1 h treatment with succinate (100 μM) or α-KG (200 μM). **(F)** Analysis of the GC surface area (*N* = 99–271 per condition) and **(G)** filopodia number (*N* = 177–187 per condition) of cortical neurons from WT, *gpr91*KO, *gpr99*KO, and double-KO mice, following a 1 h treatment with succinate (100 μM) or α-KG (200 μM). Scale bars: 5 μm. Values are presented as the means ± SEM. # indicates significant changes compared to WT in **F** and **G**; *p* < 0.001. Underlying data can be found in S1 Data. α-KG, α-ketoglutarate; E14/15, embryonic day 14/15; GC, growth cone; KO, knockout; RGC, retinal ganglion cell; WT, wild-type.
(TIF)

S3 Fig GPR91 and GPR99 ligands have no effect on RGC viability in vitro. Photomicrographs of 1 DIV embryonic mouse retinal explants **(A)** and 1 DIV embryonic cortical neurons **(B)** taken at t = 15 h after LIVE/DEAD assay experiments, in the presence or absence of succinate (100 μM), α-KG (200 μM), CI-1040 (1 μM), or Staurosporine (5 μM; positive control). CI-1040, succinate, and α-KG produced no effect on viability compared to the control. Staurosporine induced RGC cell death. Green: living cells, Red: dead cells. Scale bars: 100 μm. DIV, day in vitro; RGC, retinal ganglion cell.
(TIF)

S4 Fig GPR91 and GPR99 are not involved in guidance or target selection in vivo. **(A, B)** Lipophilic dye (DiI) tracings of the proximal visual pathway in E14/15 mouse embryos imaged with a dissecting **(A)** or a fluorescence microscope **(B)** (the red asterisk marks the location of the optic chiasm). **(A, B)** The visual pathway labeled with DiI in WT, *gpr91*KO, *gpr99*KO, and double-KO mice. The arrowhead shows the aberrant projections prior to crossing the optic chiasm, and the thin arrow highlights the fibers that have extended out of the contralateral optic tract toward the contralateral eye. **(C)** Retinogeniculate projection patterns visualized following different fluorescent CTb injections into both eyes of WT, *gpr91*KO, *gpr99*KO, and double-KO adult mice. **(D)** Quantification of the dLGN area receiving overlapping inputs (*N* = 21–42 per condition). Data are presented as the means ± SD. Scale bars: 100 μm. Underlying data can be found in S1 Data. Contra, contralateral pathway; CTb, cholera toxin subunit B; dLGN, dorsal lateral geniculate nucleus; E14/15, embryonic day 14/15; Ipsi, ipsilateral pathway; KO, knockout; ON, optic nerve; OT, optic tract; WT, wild-type.
(TIF)

S5 Fig GPR91 and GPR99 ligands have no effect on the turning of GCs from RGCs ex vivo. **(A)** Photomicrographs of time-lapse microscopy from 1 DIV mouse retinal explant GCs taken at t = 0 min and t = 60 min during GC turning assay experiments, in the presence or absence of GPR91 or GPR99 agonists. Black arrows indicate the direction of the microgradient, while blue arrowheads indicate initial GC position. Green arrowheads show the GC position following neurite growth. **(B-D)** Superimposed RGC axon trajectories over the 60-min observation period. Succinate (100 μM) increased axon growth but had no effect on the turning. α-KG (200 μM) produced no significant change on GC behavior. **(E)** Quantification of neurite elongation and GC turning responses following drug stimulation (*N* = 13–16 per condition). Scale bars: 40 μm **(A)**. Values are presented as the means ± SEM; * indicates a significant change compared to the control vehicle in **(E)**; *P* <0.0001. Underlying data can be found in S1 Data. α-KG, α-ketoglutarate; DIV, day in vitro; GC, growth cone; RGC, retinal ganglion cell.
(TIF)

S6 Fig Quantification method of retinal projection branch length and number in the DTN. Schematic representation of the method used to measure retinal projection branch length (**A**) and the number of retinal axon branches (**B**) in the DTN. Arrowed dotted lines indicate the distance between the border of the thalamus and the end of the farthest projections (A). DTN, dorsal terminal nucleus.
(TIF)

S1 Table (TIF)

## Abbreviations

ANOVA: analysis of variance
CTb: cholera toxin subunit B
DAB: 3, 3′-diaminobenzidine tetrahydrochloride
DiI: (DiIC18[3] [1,1’-dioctadecyl-3,3,3’,3’-tetramethylindocarbocyanine perchlorate])
DIV: day in vitro
dLGN: dorsal lateral geniculate nucleus
DTN: dorsal terminal nucleus
E14/15: embryonic day 14/15
ERK_1/2_: extracellular signal–regulated kinases 1 and 2
GC: growth cone
GCFL: ganglion cell fiber layer
GPCR: G-protein-coupled receptor
KO: knockout
MAPK: mitogen-activated protein kinase
NGS: normal goat serum
Oxgr1: 2-oxoglutarate receptor 1
RGC: retinal ganglion cell
S-MEM: Minimum Essential Medium Eagle Spinner Modification
Sucnr1: succinate receptor 1
TCA: tricarboxylic acid
WT: wild-type
α-KG: α-ketoglutarate
