# Supplementary figures and images for: Receptors of intermediates of carbohydrate metabolism, GPR91 and GPR99, mediate axon growth
Source: PLoS Biol. 2018 May 17;16(5):e2003619. doi: 10.1371/journal.pbio.2003619 (PMC5976209; doi:10.1371/journal.pbio.2003619)

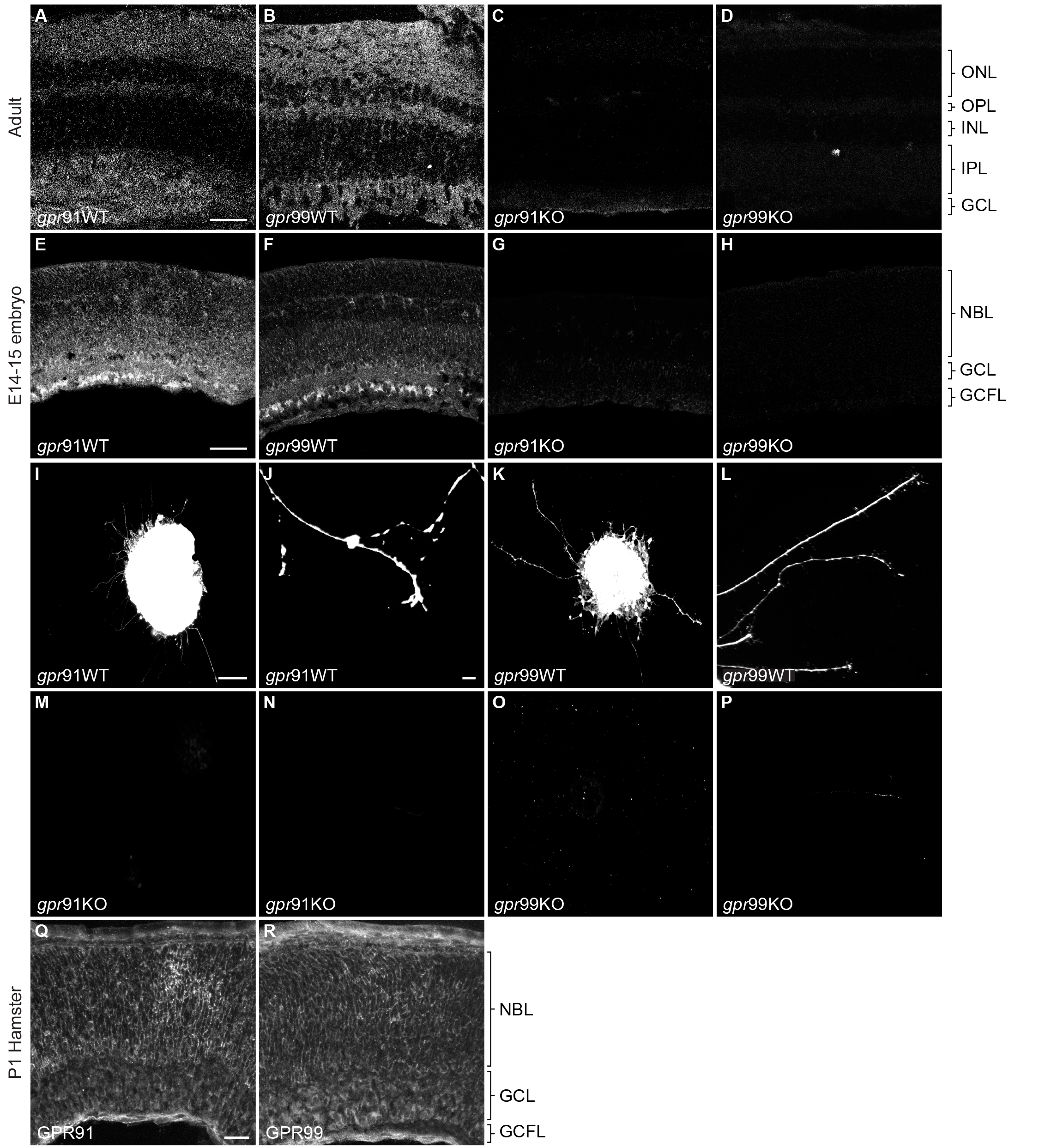

Supplement: S1 Fig — (A) Expression of GPR91 protein in the WT adult mouse retina with lack of fluorescence in the gpr91KO mouse (C). (B & D) GPR99 immunoreactivity in the retina of adult WT and gpr99KO mice. (E & G) Expression of GPR91 and (F & H) GPR99 in retinal sections of E14/15 WT, gpr91KO, and gpr99KO murine embryos. Scale bars: 75 μm (A-H). Expression of GPR91 and GPR99 in retinal explants, GCs, and neurites of E14/15 WT (I-L), gpr91KO, and gpr99KO murine embryos (M-P). Scale bars: 100 μm (I & K); 10 μm (J & L). Expression of GPR91 and GPR99 proteins in retinal sections of P1 hamster pups (Q, R). Scale bars: 10 μm. E14/15, embryonic day 14/15; GC, growth cone; GCFL, ganglion cell fiber layer; GCL, ganglion cell layer; KO, knockout; NBL, neuroblast layer; WT, wild-type. (TIF) [file pbio.2003619.s002.tif]

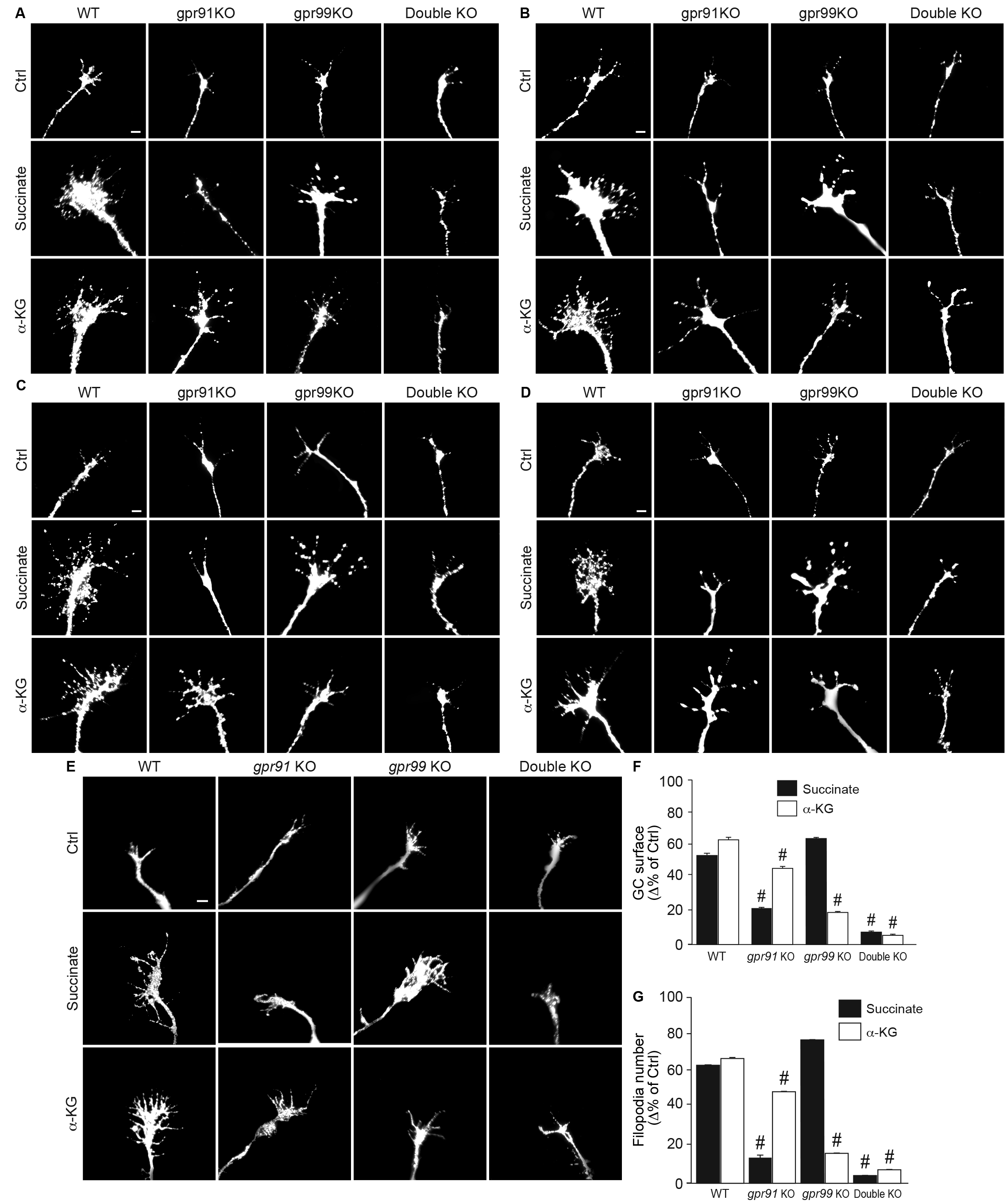

Supplement: S2 Fig — (A-D) Additional representative examples of E14/15 GCs of RGC projections from WT, gpr91KO, gpr99KO, and double-KO mouse embryos (E) Photomicrographs of E14/15 GCs of cortical neurons from WT, gpr91KO, gpr99KO, and double-KO mouse embryos after a 1 h treatment with succinate (100 μM) or α-KG (200 μM). (F) Analysis of the GC surface area (N = 99–271 per condition) and (G) filopodia number (N = 177–187 per condition) of cortical neurons from WT, gpr91KO, gpr99KO, and double-KO mice, following a 1 h treatment with succinate (100 μM) or α-KG (200 μM). Scale bars: 5 μm. Values are presented as the means ± SEM. # indicates significant changes compared to WT in F and G; p < 0.001. Underlying data can be found in S1 Data. α-KG, α-ketoglutarate; E14/15, embryonic day 14/15; GC, growth cone; KO, knockout; RGC, retinal ganglion cell; WT, wild-type. (TIF) [file pbio.2003619.s003.tif]

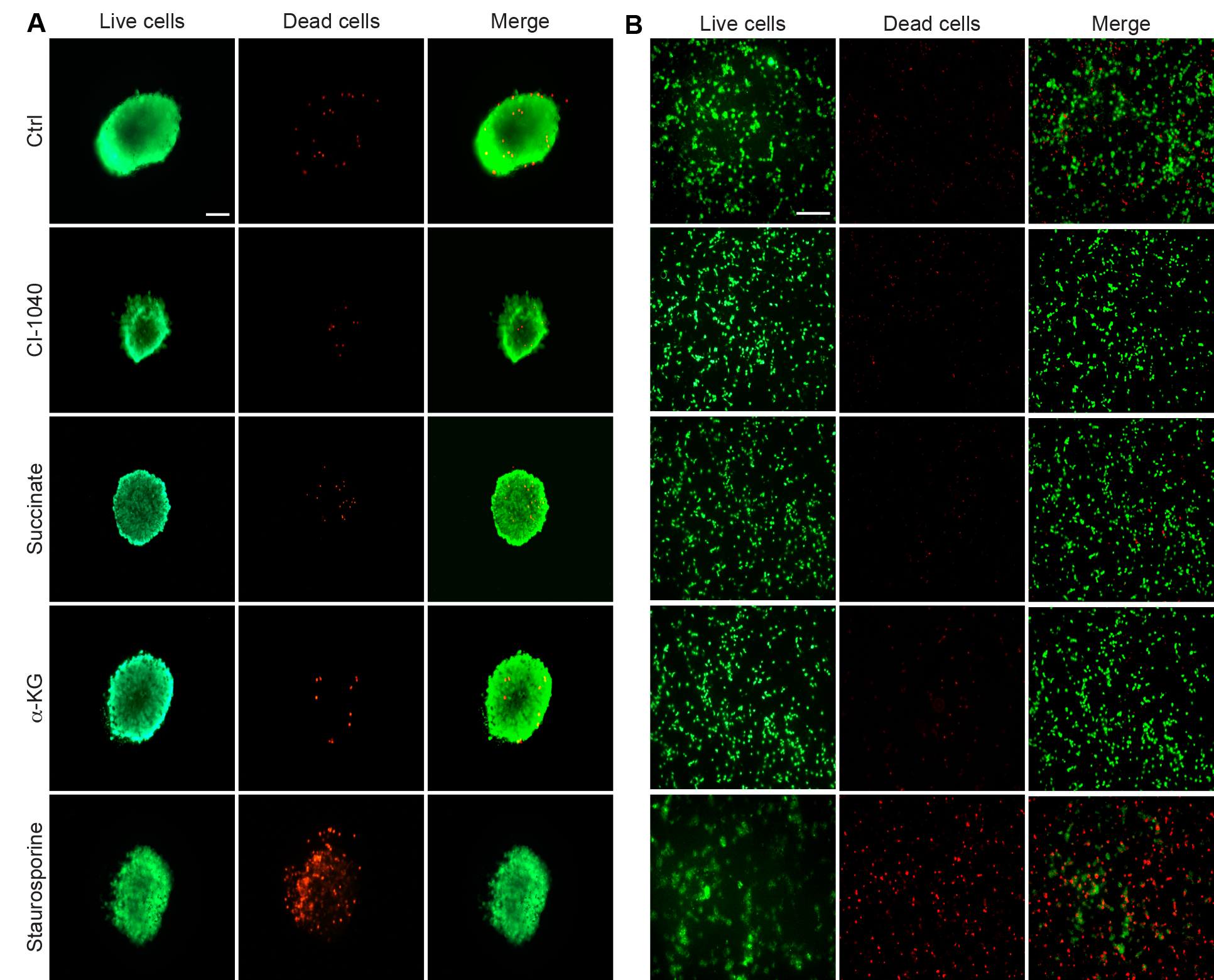

Supplement: S3 Fig — Photomicrographs of 1 DIV embryonic mouse retinal explants (A) and 1 DIV embryonic cortical neurons (B) taken at t = 15 h after LIVE/DEAD assay experiments, in the presence or absence of succinate (100 μM), α-KG (200 μM), CI-1040 (1 μM), or Staurosporine (5 μM; positive control). CI-1040, succinate, and α-KG produced no effect on viability compared to the control. Staurosporine induced RGC cell death. Green: living cells, Red: dead cells. Scale bars: 100 μm. DIV, day in vitro; RGC, retinal ganglion cell. (TIF) [file pbio.2003619.s004.tif]

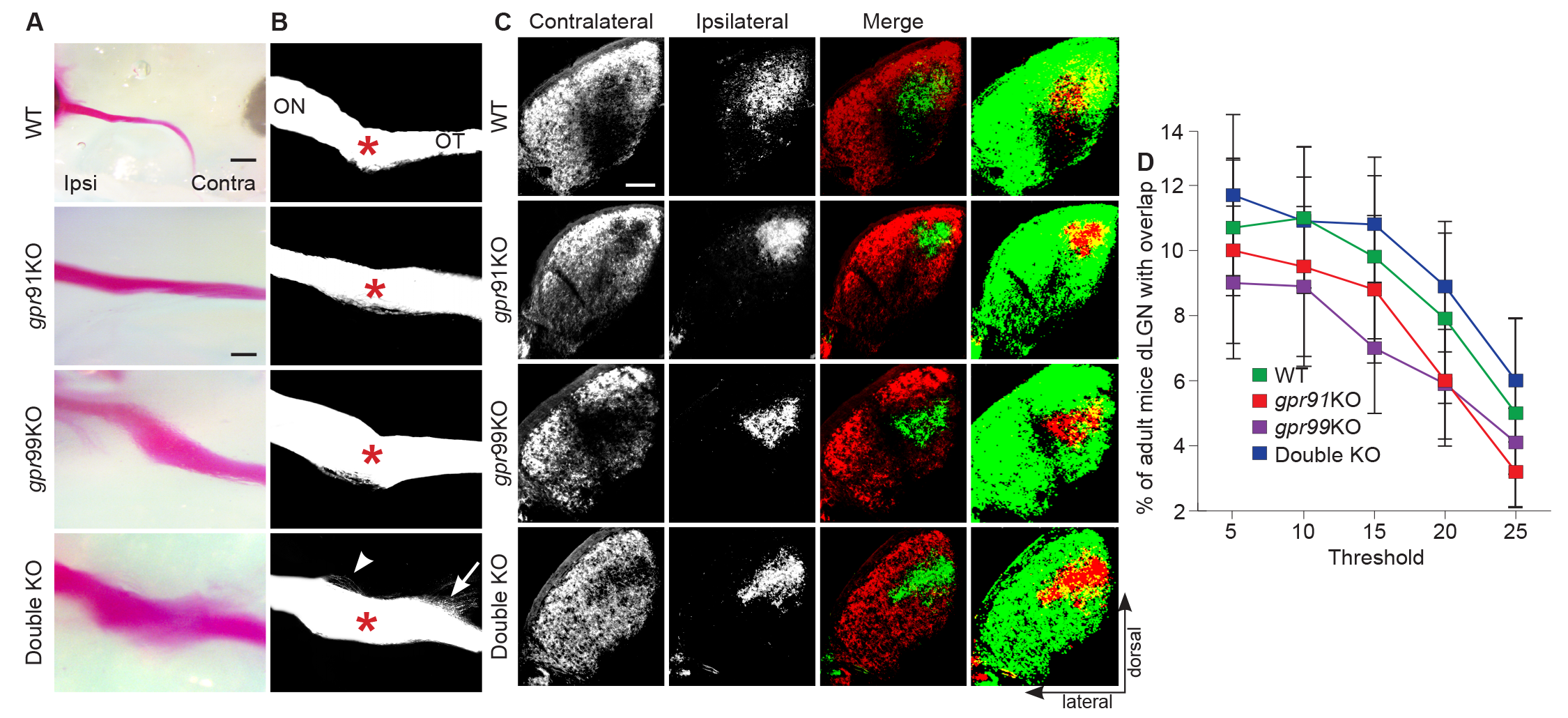

Supplement: S4 Fig — (A, B) Lipophilic dye (DiI) tracings of the proximal visual pathway in E14/15 mouse embryos imaged with a dissecting (A) or a fluorescence microscope (B) (the red asterisk marks the location of the optic chiasm). (A, B) The visual pathway labeled with DiI in WT, gpr91KO, gpr99KO, and double-KO mice. The arrowhead shows the aberrant projections prior to crossing the optic chiasm, and the thin arrow highlights the fibers that have extended out of the contralateral optic tract toward the contralateral eye. (C) Retinogeniculate projection patterns visualized following different fluorescent CTb injections into both eyes of WT, gpr91KO, gpr99KO, and double-KO adult mice. (D) Quantification of the dLGN area receiving overlapping inputs (N = 21–42 per condition). Data are presented as the means ± SD. Scale bars: 100 μm. Underlying data can be found in S1 Data. Contra, contralateral pathway; CTb, cholera toxin subunit B; dLGN, dorsal lateral geniculate nucleus; E14/15, embryonic day 14/15; Ipsi, ipsilateral pathway; KO, knockout; ON, optic nerve; OT, optic tract; WT, wild-type. (TIF) [file pbio.2003619.s005.tif]

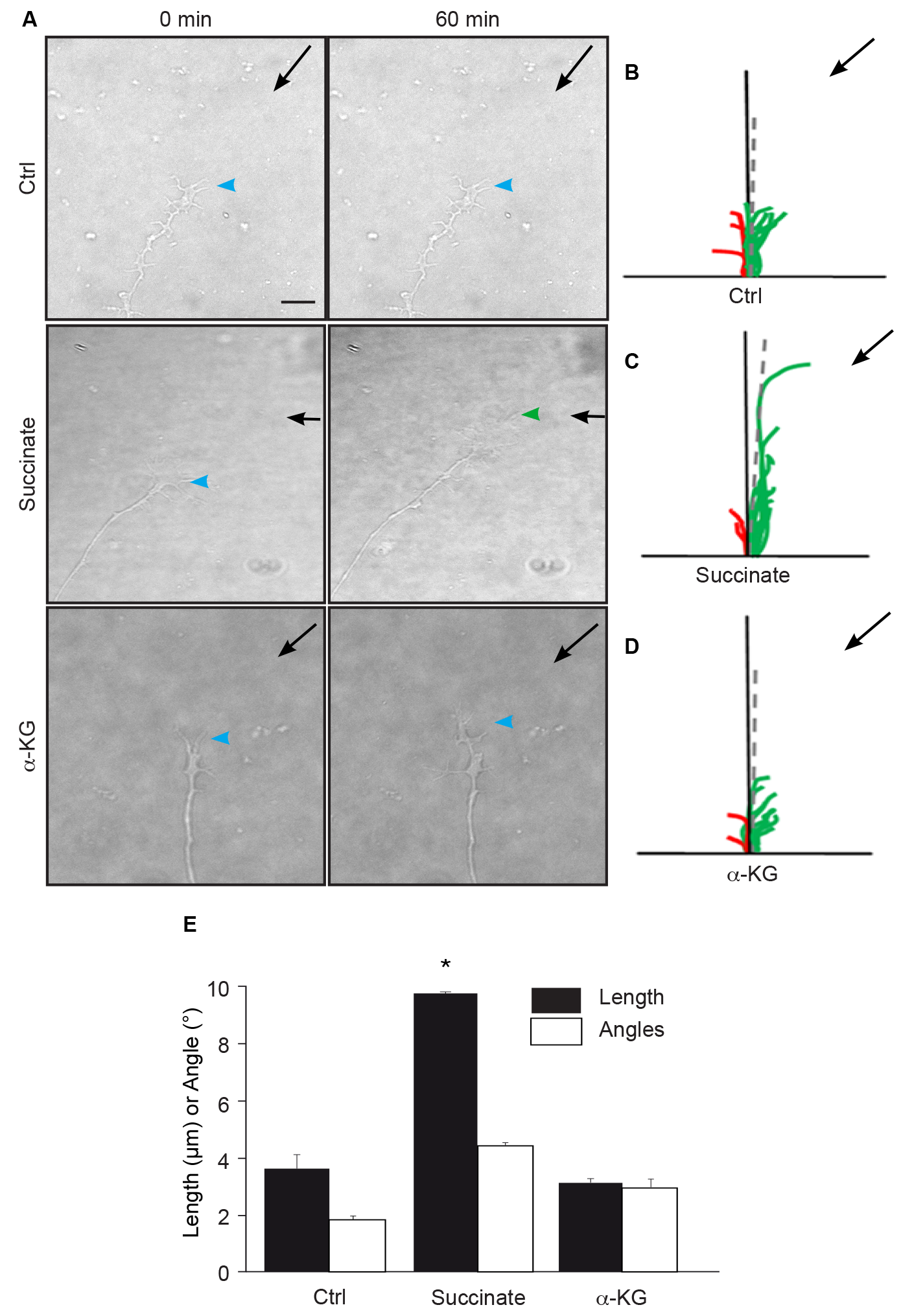

Supplement: S5 Fig — (A) Photomicrographs of time-lapse microscopy from 1 DIV mouse retinal explant GCs taken at t = 0 min and t = 60 min during GC turning assay experiments, in the presence or absence of GPR91 or GPR99 agonists. Black arrows indicate the direction of the microgradient, while blue arrowheads indicate initial GC position. Green arrowheads show the GC position following neurite growth. (B-D) Superimposed RGC axon trajectories over the 60-min observation period. Succinate (100 μM) increased axon growth but had no effect on the turning. α-KG (200 μM) produced no significant change on GC behavior. (E) Quantification of neurite elongation and GC turning responses following drug stimulation (N = 13–16 per condition). Scale bars: 40 μm (A). Values are presented as the means ± SEM; * indicates a significant change compared to the control vehicle in (E); P <0.0001. Underlying data can be found in S1 Data. α-KG, α-ketoglutarate; DIV, day in vitro; GC, growth cone; RGC, retinal ganglion cell. (TIF) [file pbio.2003619.s006.tif]

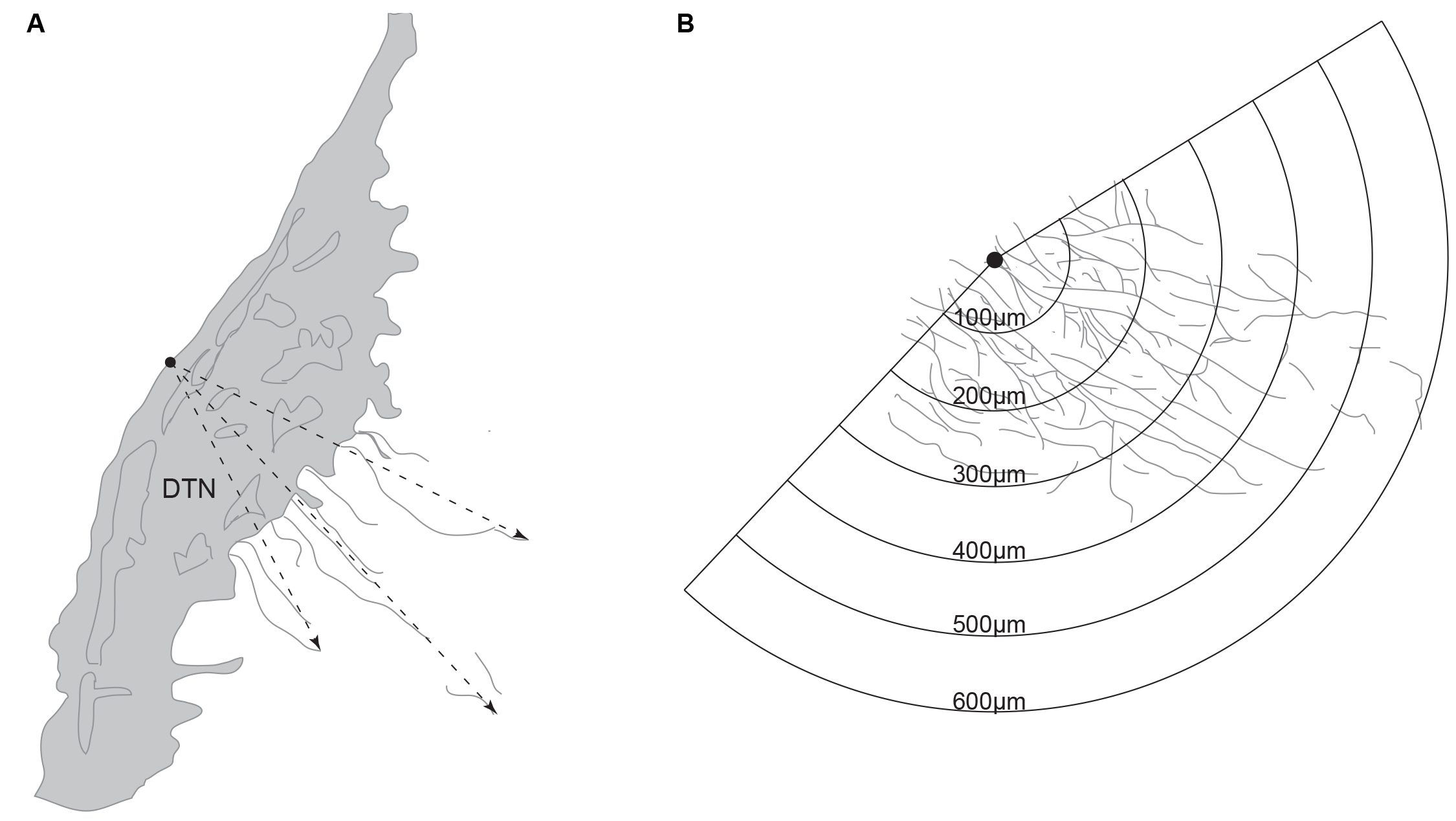

Supplement: S6 Fig — Schematic representation of the method used to measure retinal projection branch length (A) and the number of retinal axon branches (B) in the DTN. Arrowed dotted lines indicate the distance between the border of the thalamus and the end of the farthest projections (A). DTN, dorsal terminal nucleus. (TIF) [file pbio.2003619.s007.tif]

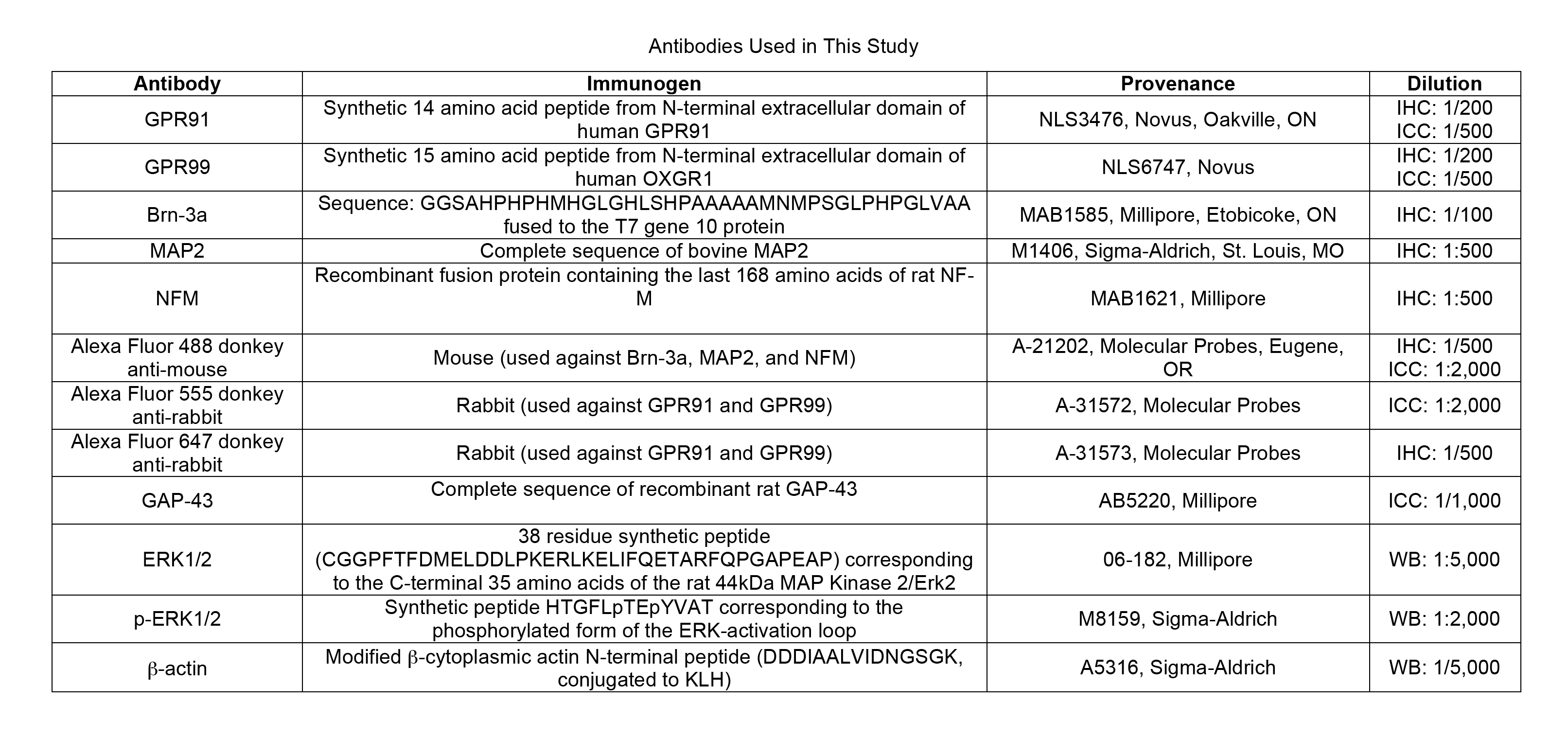

Supplement: S1 Table — (TIF) [file pbio.2003619.s008.tif]
